# RNA-seq reveals differentially expressed genes involved in catecholamine synthesis and metabolism in the hypothalamus affecting divergent residual feed intake in slow-growing Korat chickens

**DOI:** 10.1016/j.psj.2026.107098

**Published:** 2026-05-08

**Authors:** Panpradub Sinpru, Orapin Jantasaeng, Phocharapon Pasri, Boonyarit Kamkrathok, Saknarin Pengsanthia, Tom E. Porter, Wittawat Molee, Amonrat Molee

**Affiliations:** a School of Animal Technology and Innovation, Institute of Agricultural Technology, Suranaree University of Technology, Nakhon Ratchasima 30000, Thailand; b Department of Animal and Avian Sciences, University of Maryland, College Park, MD 20742, United States

**Keywords:** Hypothalamus, Feed efficiency, Residual feed intake, RNA-seq, Slow-growing chicken

## Abstract

Korat chicken (**KRC**), a slow-growing chicken known for its unique meat flavor and nutritional value, exhibits variation in feed efficiency (**FE**). Since the hypothalamus acts as a convergent and integrative center for multiple nutrient-related signals, this study aims to compare transcriptomic profiles and neuronal pathways in hypothalamus between two groups of male KRCs differing in residual feed intake (**RFI**). RNA was extracted from hypothalamic tissues of males KRC either in the low-RFI (*n* = 10) or in the high-RFI (*n* = 10) group. The results showed 257 DEGs, including 138 upregulated and 119 downregulated genes in the low RFI compared to the high-RFI groups. Gene Ontology analysis of the DEGs revealed that they were mainly related to metabolic processes and transporter activity. Kyoto Encyclopedia of Genes and Genomes pathway analysis identified 3 significant pathways, including the folate biosynthesis (3 genes including *GCH1, TPH2*, and *TH*), tyrosine metabolism (3 genes including *DDC, TH* and *FAH*), and tryptophan metabolism (3 genes including *DDC, TPH2*, and *TDO2*) pathways. The upregulated genes in the low-RFI group (*TPH2, DDC*, and *TH*) were enriched in the folate biosynthesis, tyrosine metabolism, and tryptophan metabolism pathways, which is consistent with the observed plasma concentrations of dopamine (**DA**) and serotonin/5-hydroxytryptamine (**ST/5-HT**). These findings suggest that differences in DA and ST/5-HT levels among the chicken groups may be associated with variation in FE. Furthermore, genes such as *GCH1, TPH2, DDC, TH,* and *FAH* could be candidate genes supporting the role of hypothalamus in regulating FE of slow-growing KRC.

## Introduction

Korat chicken (**KRC**) is a crossbreed between a male Thai indigenous chicken line (Leung Hang Khao, **LK**) and a female broiler line (Suranaree University of Technology, **SUT**). KRC is classified as a slow-growing chicken, with an average daily gain (**ADG**) of 24.13 g/days, reaching a body weight (**BW**) of 1.79 kg after 12 weeks of age, and achieving a feed conversion ratio (**FCR**) of 2.94 (Hang et al., 2018). As feed costs represent approximately 60–70 % of total poultry production expenses, feed utilization is a critical determinant of profitability in the poultry industry (Guo et al., 2024). To sustain the income of smallholder farmers and reduce the ecological footprint, it is essential to improve feed efficiency (**FE**) of KRC.

FE in animals depends on the relationship between their feed intake (**FI**) and growth, wherein the brain integrates metabolic signals from peripheral tissues and subsequently coordinates adaptive changes in FI and energy expenditure (Blouet and Schwartz, 2010). In KRC, high-throughput omics technology has been used to compare gene expression patterns between animals differing in FE, providing candidate biomarkers and identifying mechanisms to enhance FE through genetic selection. Results obtained on the jejunum identified differentially expressed genes (**DEGs**) enriched in pathways related to immune response, glutathione metabolism, vitamin transport and metabolism, lipid metabolism, neuronal and cardiac maturation, development, and growth (Sinpru et al., 2021). A similar study on the duodenum showed that high FE chickens exhibited better glucose breakdown for energy extraction through glycolysis and stronger tight junctions in their intestinal epithelium as compared to low FE chickens (Kaewsatuan et al., 2022). Furthermore, our previous results identified several DEGs between the low-RFI and high-RFI KRC in pituitary gland tissue that may contribute to their phenotypic divergence, such as regulator of G-protein signaling 1 (***RGS1***), thyrotropin-releasing hormone receptor3 (***TRHR3***), solute carrier family 6 member 1 (***SLC6A1***), solute carrier family 6 member 13 (***SLC6A13***), solute carrier family 13 member 3 (***SLC13A3***), PR/SET domain 6 (***PRDM6***), ATPase 13A5 (***ATP13A5***), and inter-alpha-trypsin inhibitor heavy chain 2 (***ITIH2***). These genes are associated with metabolic homeostasis, energy balance, nutrient metabolism, and hormone-regulated signaling pathways (Sinpru et al., 2025). However, the regulatory mechanisms underlying the hypothalamic response to differences in FE are complex and remain unclear, particularly in slow-growing KRCs.

The hypothalamus plays a central role in regulating food intake and energy expenditure (Sohn, 2015), it functions as a critical brain region that integrates metabolic signals from peripheral tissues to maintain FI and energy balance under changing metabolic conditions (Blouet and Schwartz, 2010). Catecholamines (like dopamine, norepinephrine) are crucial neurotransmitters in the hypothalamus, a brain region controlling hormones and bodily functions; they regulate appetite, stress response, cardiovascular control, and reproductive hormones by acting on specific hypothalamic nuclei and influencing the pituitary gland, linking the nervous system to the endocrine system, with varying levels responding to stress, hydration, and even leptin signaling (Neill et al., 1979). It has been reported that hypothalamic neuropeptide gene expression [neuropeptide Y (***NPY***) receptor Y5] influences FE in ducks (Zeng et al., 2016) by decreasing FI and increasing anorexia in the group exhibiting low residual feed intake (**RFI**). A study on gene expression in the hypothalamus of pigs reported that DEGs between low and high RFI individuals were enriched in pathways related to neuronal signaling that regulates FI, such as the feeding behavior pathway and the olfactory bulb development pathway (Hou et al., 2018). In ducks, an increase in gamma-aminobutyric acid (**GABA**) type A receptors (gamma-aminobutyric acid type A receptor subunit delta and gamma-aminobutyric acid type A receptor subunit beta 1) was reported in the high-RFI group, which may activate GABA release through the GABA signaling pathway, resulting in the activation of the arcuate nucleus NPY/agouti-related peptide neurons to elicit feeding responses (Guo et al., 2024). In laying hens, selection for different RFI values was associated with the expression of neuropeptides such as hypothalamic suppressor of cytokine signaling 3, ghrelin receptor, NPY, and agouti-related peptide in the high-RFI line (Sintubin et al., 2014). Based on these findings, we hypothesized that changes in gene expression at the hypothalamic level may activate essential molecular processes that contribute to FE in chickens. However, no studies have reported the hypothalamic neuronal signaling transduction-related genes associated with FE variation in slow-growing chickens. This is the first study to elucidate the transcriptomic profile and pathway related to FE in hypothalamus in slow-growing KRC. Thus, this study aimed to identify candidate genes and pathways associated with neuronal signal transduction underlying differences in RFI between low- and high-RFI groups using RNA sequencing (RNA-seq). The findings from this study can be applied as a model for slow-growing chickens to elucidate the hypothalamic neuronal signaling mechanisms underlying FE, a key economic trait that impacts production costs and contributes to environmental sustainability.

## Materials and methods

### Ethics statement

The experimental procedures and protocols applied in this study were approved by the Ethics Committee on Animal Use of Suranaree University of Technology, Nakhon Ratchasima, Thailand (SUT-IACUC-0016/2022).

### Animals, housing, feed efficiency assessment, and tissue sampling

To produce KRC, 20 LK males and 100 SUT females were mated at a ratio of 1:5. All one-day-old KRC chicks were vent-sexed, and a total of 115 male KRC chicks were wing-banded and individually weighed at hatch. The chicks were housed in individual cages and provided *ad libitum* access to commercial feed (CPF Co., Ltd., Nakhon Ratchasima, Thailand), a starter diet until 3 weeks of age (3,946 kcal/kg of gross energy and 21 % protein), a grower diet from 4 to 6 weeks of age (4,081 kcal/kg of gross energy and 19 % protein), and a finisher diet (4,111 kcal/kg of gross energy and 17 % protein) from 7 to 10 weeks of age. BW and FI of the KRC were recorded individually from 1 to 10 weeks of age. At 10 weeks of age, RFI was calculated as described by Poompramun et al. (2021). The formula for RFI calculation is shown below:

$$RFI_{j}=Total\;feed\;intake\;for\;week_{j\;\left(g\right)}-\left(b_{0}+b_{1}MMW_{j}+b_{2}BWG_{j}\right)$$

where *BWGj* was the BW gain (g) during *week_j_* and *MMW_j_* was the metabolic weight estimated from the mean BW at *week_j_*, i.e.,

$${\left(\frac{\mathrm{Init}\mathrm{ial}\;\mathrm{body}\;\mathrm{weig}\mathrm{ht}+\;\mathrm{Final}\;\mathrm{body}\;\mathrm{weig}\mathrm{ht}}{2}\right)}^{0.75}$$

were *b*_0_ was the intercept, and *b*_1_ and *b*_2_ are partial regression coefficients. Then cumulative RFI from hatch to week _j_ was calculated as below:

$$RFIj=\sum_{i=1}^{j}RFIi$$

Based on RFI values at 10 weeks of ages, 10 chickens with extremely low RFI and 10 chickens with extremely high RFI were identified, forming the low-RFI and high-RFI groups, respectively. Prior to slaughter, all 20 birds were subjected to an 8 h feed withdrawal but had constant access to water. After electrical stunning (150 mA, frequency of 200 Hz for 4 s), the chickens were euthanized by decapitation, and hypothalamic samples were carefully dissected with the aid of an illuminated magnifying lens. Initial incisions were made just anterior to the oculomotor nerve (nervus oculomotorius) and posterior to the tuberculum olfactorium, based on published descriptions and diagrams (Kuenzel and Masson, 1988). Next, lateral cuts were made approximately 2 mm from the midline to yield a rectangular piece of tissue. This was placed on its side, and a final cut was made at a depth immediately below the subseptal organ (organum subseptale) and parallel to the basal surface of the hypothalamus. This dissection protocol consistently captured a core region of feeding-related nuclei, predominantly the infundibular nucleus (**IN**), ventromedial nucleus (**VMH**), and paraventricular nucleus (**PVN**) (Boswell et al., 2002; Chen et al., 2007; Kosonsiriluk et al., 2008). The hypothalamus was collected into tubes, immediately frozen in liquid nitrogen, and stored at −80°C until further RNA extraction analysis. Then, abdominal fat was carefully dissected, collected, and subsequently weighed.

### Total RNA extraction and quality control

The total RNA of hypothalamic samples was isolated from the 20 male chickens (10 chickens from each treatment group) using TRIzol Reagent (Thermo Fisher Scientific, Waltham, MA) according to the manufacturer’s protocol. RNA concentration was measured using a NanoDrop 2000 spectrophotometer (Thermo Fisher Scientific, Waltham, MA). To increase RNA yield and minimize variation arising from tissue dissection, equal amounts of RNA from two individual birds within each group were pooled to generate one biological replicate, resulting in five pooled samples per group for the low-RFI and high-RFI treatments. RNA pools were constructed based on ranked RFI values, whereby samples from birds with adjacent RFI rankings within the same phenotypic group were combined to form each pooled sample. RNA purity of the pooled samples was verified using 1 % agarose gel electrophoresis, and the RNA integrity number (**RIN**) was assessed using the 5400 Fragment Analyzer Systems (Agilent Technologies, Santa Clara, CA). One µg of total RNA with RIN ≥ 7.7 was used for cDNA library construction. The remaining RNA was kept at −80°C for validation assays.

### cDNA library construction, and RNA-seq

cDNA libraries preparation and RNA-seq were performed by the Novogene Biotechnology Company (Novagene, Beijing, China), according to the manufacturer’s user guide (Illumina, San Diego, CA). A total of 1 µg RNA per pooled sample was used for the construction of 10 libraries. Briefly, mRNA was purified from total RNA using poly-T oligo-attached magnetic beads. After fragmentation, the first strand cDNA was synthesized using random hexamer primers, and the second strand cDNA was subsequently performed using dUTP for a directional library. The library fragments were purified with the AMPure XP system (Beckman Coulter, Beverly, USA), and cDNA fragments of 370-420 bp in length were selected for PCR amplification to create the final cDNA library. The libraries were sequenced on an Illumina Novaseq 6000 instrument (Illumina, San Diego, CA) with 150 bp paired end reads method.

### Bioinformatics analysis

Raw reads of FASTQ format were filtered to remove the adapter and low-quality reads (Martin, 2011). The Phred score Q20, Q30, and GC content were calculated simultaneously. High-quality clean reads from 10 pooled samples were mapped to the chicken reference genome (GRCg6a, GenBank assembly accession: GCA_000002315.5) using the Hisat2 version 2.0.5 software (Kim et al., 2013). To identify DEGs between the low-RFI and high-RFI groups, raw read counts were used as input for the DESeq2 R package (version 1.20.0) (Love et al., 2014). Gene expression levels were also estimated as reads per kilobases per million reads (**RPKM**) for comparative purposes. The thresholds for DEGs were set at *P*-value < 0.05 and |log 2-fold change| > 1. Functional enrichment analysis of DEGs, including Gene Ontology (**GO**) and Kyoto Encyclopedia of Genes and Genomes (**KEGG**) pathways, was conducted using the clusterProfiler R package (version 3.8.1), and a *P*-adjusted value (*P*-adj) < 0.05 was considered significantly enriched. Protein-protein interaction network analysis of DEGs was performed using the STRING database (version 10; http://string-db.org/).

### Quantitative real-time PCR validation

To validate the reproducibility and accuracy of the RNA-seq data between the low RFI and high RFI groups, reverse transcription-quantitative PCR (**RT-qPCR**) was performed using the same five pooled RNA samples per group. Five DEGs were selected for validation, including l-DOPA decarboxylase *(****DDC***), tyrosine hydroxylase (***TH***), tryptophan hydroxylase 2 (***TPH2***), fumarylacetoacetate hydrolase (***FAH***), and GTP cyclohydrolase 1(***GCH1***), based on their high connectivity and involvement in the regulatory network influencing feeding behavior. RT**-**qPCR was conducted on a LightCycler 480 System (Roche Diagnostics GmbH, Mannheim, Germany) using SYBR Green Master Mix (Thermo Fisher Scientific, Carlsbad, CA). The thermal cycling conditions were 95°C for 10 min, followed by 40 cycles of 95°C for 30 s, 60°C for 1 min and 72°C for 30 s. Primer sequences were designed using Ensembl (https://ensembl.org) and are provided in Table S1. Five pooled RNA samples were analyzed per condition, with each pooled sample measured in technical triplicate. Relative gene expression was calculated using the 2^−∆∆Ct^ method (Livak and Schmittgen, 2001), with β-actin as the reference gene. The expression values of each gene were log_2_-transformed to facilitate comparison between the RT–qPCR and RNA–seq results.

### Validation of enriched KEGG pathway of DEGs

To validate the key metabolic and signaling pathways identified through our transcriptomic analysis, we performed targeted analyses to support the biological relevance of these findings. Blood was collected from ten birds in each group and placed in EDTA tubes, then centrifuged at 4,000 × *g* for 15 min at 4°C. Plasma samples were aliquoted into 1.5 mL tubes and stored at −20°C for subsequent KEGG pathway validation analysis. Dopamine (**DA**) levels were measured using a DA ELISA kit (Antibodies.com LLC, MO, USA), and serotonin/5-hydroxytryptamine (**ST/5-HT**) levels were measured using a competitive ELISA kit (Thermo Fisher Scientific, Bender MedSystems GmbH, Vienna, Austria). A four-parameter logistic (4-PL) curve fit was created as a standard curve for calculating sample concentrations.

### Statistical analysis

The differences in mean phenotype, gene expression levels and plasma levels of DA and ST/5-HT between low RFI and high RFI groups was assessed using Student’s t-test in the SPSS 24.0 software package (SPSS Inc., Chicago, IL). *P*-value < 0.05 was considered statistically significant. All data were expressed as mean ± SEM.

## Results and discussion

### Phenotypic measurements

The KRC with extreme RFI phenotypes showed significant differences in FI, FCR, and RFI (*P* < 0.01), but there were no significant differences in BW, BWG, ADG, and abdominal fat (*P* > 0.05) (Table 1). Chickens with low-RFI group exhibited significantly lower FI, FCR, and RFI than high-RFI group (*P* < 0.01). The RFI value of the low-RFI group was −233.98 ± 12.23 g compared with 256.53 ± 21.90 g for the high-RFI group. As expected from the definition of RFI, KRC in the low-RFI group showed higher FE with lower FI while BW, BWG, ADG, and abdominal fat were not affected.

**Table 1:** Phenotypic traits of the low-RFI and high-RFI groups (mean ± SEM).

Trait	Low-RFI (*n* = 10)		High-RFI (*n* = 10)		Mean difference (Low-High), 95 % CI	*P-value*
	Mean ± SEM	CV (%)	Mean ± SEM	CV (%)		
BW (g)	1,388.80 ± 36.72	8.36	1,456.00 ± 26.48	5.75	−67.20 (−155.90, 21.50)	0.16
BWG (g)	1,341.40 ± 36.47	8.60	1,411.50 ± 26.53	5.94	−70.10 (−158.70, 18.50)	0.14
ADG (g/d)	19.30 ± 0.51	8.36	20.20 ± 0.38	5.95	−0.90 (−2.08, 0.28)	0.17
FI (g)	2,977.00 ± 66.64	7.08	3,560.60 ± 62.37	5.54	−583.60 (−783.30, −383.90)	<0.01
FCR	2.22 ± 0.20	28.49	2.52 ± 0.20	25.10	−0.30 (−0.89, 0.29)	<0.01
RFI (g)	−233.98 ± 12.23	16.53	2,56.53 ± 21.90	27.00	−490.51 (−545.40, −435.60)	<0.01
Abdominal fat (g)	11.61 ± 2.27	61.83	15.15 ± 2.45	51.14	−3.54 (−10.70, 3.60)	0.30
Abdominal fat (%)	0.83 ± 0.16	60.96	1.04 ± 0.18	54.73	−0.21 (−0.68, 0.26)	0.38

The mean values of phenotypic traits were calculated from individual birds (*n* = 10 per group). CV: coefficient of variation. Mean differences were calculated as Low RFI-High RFI. The 95 % confidence intervals (CI) were estimated based on group means and standard deviations. Differences between groups were assessed using a Student’s t-test. RFI: residual feed intake; BW: body weight at 10 weeks; BWG: body weight gain from 1 to 10 weeks; ADG: average daily gain; FI: total feed intake from 1 to 10 weeks; FCR: feed conversion ratio.

Regarding abdominal fat, no significant differences were observed between the low-RFI and high-RFI groups. Abdominal fat is generally considered an indicator of energy metabolism in meat-type chickens (Baéza and Le Bihan-Duval, 2013), and its percentage has shown positive phenotypic correlations with RFI in both broilers (Zhuo et al., 2015; Li et al., 2020; Chen et al., 2021a) and native slow-growing chickens. A crossbreeding experiment conducted in Ethiopia between a commercial slow-growing line (SASSO line) and native chickens (Koekoek) showed significant differences in BW and in FE between crossbred and purebred but no difference in abdominal fat percentage, with moderate values (0.9 to 0.8 % in crossbred) (Itafa et al., 2021). The present study includes only crossbred chickens with a slow-growing genetic background, which exhibited a level of abdominal fat percentage quite similar to the one observed by Itafa et al. (2021). This suggests that FE may not directly influence abdominal fat deposition in crossbred chickens, which may have unique metabolic characteristics compared to pure lines of broilers or indigenous chickens. Furthermore, the relationship between FE and abdominal fat percentage in crossbreds may be affected by heterosis effects that remain to be studied.

In this study, KRC chickens classified into low-RFI and high-RFI groups exhibited clear divergence in FE traits. Collectively, these findings indicate that improved FE (low RFI) is primarily driven by FI rather than differences in growth performance, providing a physiological basis for subsequent molecular analyses of metabolic and neuroendocrine regulation.

### Quality of hypothalamic RNA-seq data

The results of the RNA-seq and mapping data are shown in Table S2. An average of 41,286,507 clean reads were generated in the hypothalamic tissue. The clean reads were mapped to the *Gallus gallus* −6.0 genome with a total map ranging from 90.14 % to 91.70 %. The average GC content of the samples was approximately 47.64 %, while the average percentages of Q20 and Q30 bases were 96.24 % and 90.80 %, respectively. According to gene expression levels of all pooled samples, the heat map showed a high correlation between the samples (values greater than 0.90) (Fig. S1).

The transcriptome sequencing data are available through the Sequence Read Archive (**SRA**) of NCBI under accession number GSE278826.

### Pooling rationale and implications

In this study, a pooling strategy was adopted to balance statistical power with practical constraints, including limited resources and RNA yield, while ensuring the ethical use of animals. This pooling strategy is a cost-effective approach that has been widely applied in comparable RNA-seq studies (Izadnia et al., 2019; Kubota et al., 2023; Teufel and Sobetzko, 2022). Furthermore, when pooled samples are representative, balanced, and processed uniformly, pooling is not expected to introduce systematic bias in the direction or magnitude of group-level differences (Takele Assefa et al., 2020). Nevertheless, the resulting transcriptomic profiles reflect average group-level expression patterns and should therefore be interpreted at the population level rather than as indicators of individual-level responses.

In addition, RT-qPCR validation was performed using the same pooled RNA samples employed for RNA-seq analysis. Thus, these results provide technical confirmation of sequencing consistency rather than independent biological validation at the individual-animal level. The detailed RNA pooling scheme, including the assignment of individual birds to each pooled sample, has now been provided in Table S3.

### Differentially expressed genes

**Fig. 1:**
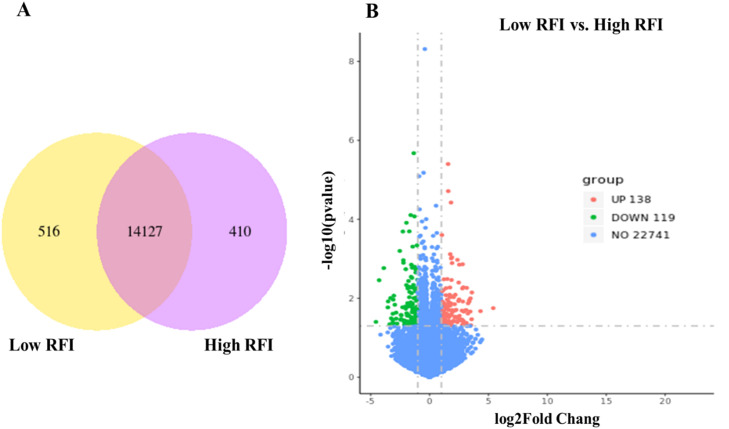
Venn diagram shows the number of genes expressed (A) and volcano plots (B) of differentially expressed genes (DEGs) in hypothalamic tissues between low-RFI and high-RFI groups.

A total of 15,053 genes were identified in the hypothalamus with RPKM > 1. Out of the identified genes, 516 and 410 were unique for low-RFI and high-RFI groups, respectively, and 14,127 were co-expressed in both groups (Fig. 1A). After comparison, 257 genes were considered to be significant DEGs between low-RFI and high-RFI groups under the *P*-value < 0.05 and |log 2-fold change| > 1. Among these DEGs, 138 were upregulated and 119 were downregulated (Fig. 1B), and their expression values are provided in Table S4. The results of the DEG analysis support our hypothesis that variation in FE is reflected in gene expression at the hypothalamic level. A detailed discussion of these results is provided in the following section.

### GO classification of DEGs

**Fig. 2:**
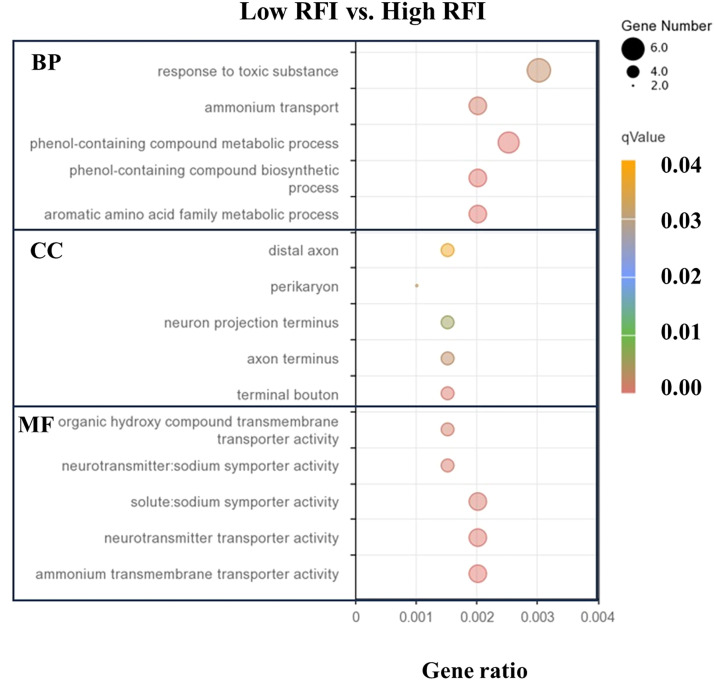
Top 15 gene ontology (GO) enrichment analyses of differentially expressed genes (DEGs) in the hypothalamic tissues. BP, Biological process; CC, Cellular component; MF, Molecular function. Terms were detected from DEGs in the hypothalamic tissues of low-RFI compared to high-FRI group.

To understand the functional classification of DEGs, GO and KEGG pathway analyses of the 257 DEGs were conducted. A total of 113 significantly enriched terms were identified, belonging to the categories of biological process (**BP**), cellular component (**CC**), and molecular function (**MF**) (Table S5). The DEGs were mainly enriched in the GO terms with BP and MF categories between low-RFI and high-RFI groups (Fig. 2). Among the BP terms, most DEGs were related to response to toxic substance (GO:0009636), ammonium transport (GO:0015696), phenol-containing compound metabolic process (GO:0018958), phenol-containing compound biosynthetic process (GO:0046189), and aromatic amino acid family metabolic process (GO:0009072). The largest number of DEGs in the MF terms were related to solute:sodium symporter activity (GO:0015370), neurotransmitter transporter activity (GO:0005328), and ammonium transmembrane transporter activity (GO:0008519).

Interestingly, we found that the DEGs were mainly related to metabolic processes and transporter activity between low-RFI and high-RFI groups. Given that the hypothalamus plays a central role in appetite regulation by integrating, coordinating, and transmitting nutrient-related signals from both peripheral and central systems (Liu et al., 2019), these metabolic processes within hypothalamic tissue are likely to contribute to the modulation of feeding behavior in poultry (Guo et al., 2024). Such regulation occurs through interactions among neuronal hormones, receptors, and substrate sensors, which may influence feeding responses (Seong et al., 2019). Studies on chickens have shown that neuroactive ligand-receptor interactions control food intake and energy expenditure by coordinating multiple neural networks (Li et al., 2018). Peripheral neuropeptides, including cholecystokinin (**CCK**), ghrelin, and peptide YY (**PYY**), provide feedback to the central nervous system by regulating gastrointestinal functions such as motility, secretion, and absorption, thereby playing a crucial role in appetite control (Arora and Anubhuti, 2006). This finding aligns with previous studies indicating that the neuroactive ligand-receptor interaction pathway involves all receptors and ligands on the plasmalemma that are related to intracellular and extracellular signaling pathways (Mao et al., 2005). These results suggest that changes in hypothalamic gene expression between low-RFI and high-RFI groups may influence metabolism by modulating the neuroendocrine regulations of energy balance (Guo et al., 2024). Collectively, these molecular differences likely affect feeding behavior, neuroendocrine signaling, and overall metabolic homeostasis, thereby contributing to the distinct FE phenotypes observed between the two groups.

### KEGG pathway enrichment of DEGs

To further elucidate the functional roles of the identified DEGs, KEGG pathway enrichment analysis was conducted. Six DEGs were significantly enriched in three pathways related to neuronal signaling and the regulation of FE (*P-adj < 0.05*) when comparing the low-RFI and high-RFI groups: folate biosynthesis, tyrosine metabolism, and tryptophan metabolism, as shown in Table 2.

**Table 2:** Significantly enriched KEGG pathway of differentially expressed genes (DEGs) in the hypothalamic tissues between low-RFI and high-RFI groups.

KEGG ID	Description	*P*-value	*P*-adj	Gene name^1^	Count
gga00790	Folate biosynthesis	0.001314	0.036393	*GCH1↑/TPH2↑/TH↑*	3
gga00350	Tyrosine metabolism	0.001897	0.036393	*DDC↑/TH↑/FAH↑*	3
gga00380	Tryptophan metabolism	0.002426	0.036393	*DDC↑/TPH2↑/TDO2*↑	3

1 *GCH1*: GTP cyclohydrolase 1; *TPH2*: tryptophan hydroxylase 2; *TH*: tyrosine hydroxylase; *DDC*: l-DOPA decarboxylase; *FAH*: fumarylacetoacetate hydrolase; *TDO2*: tryptophan 2,3-dioxygenase. Up and down arrows indicate the upregulated and downregulated genes, respectively, in hypothalamic tissues when comparing the low-RFI and high-RFI groups.

Folate (vitamin B9) is essential for the metabolism of nucleic acid precursors and several amino acids, as well as for methylation reactions (Tola, 2024). It has been reported that folate can enhance the proliferation of adipocytes while inhibiting lipid deposition in chicken preadipocytes (Yu et al., 2014). Moreover, folate can reduce triglyceride deposition in primary chicken hepatocytes, potentially by affecting antioxidant function and lipid metabolism (Liu et al., 2018). In rats, alterations in folate may affect feeding mechanisms in the hypothalamus, as it modifies the expression of feeding-related neuropeptide genes (Cho et al., 2013). In this study, some DEGs in hypothalamic tissues between low-RFI and high-RFI groups were enriched in the folate biosynthesis pathway, which is an important pathway related to FE in pigs (Banerjee et al., 2020). *GCH1* is the first catalyzing and rate-limiting enzyme controlling metabolic flux through the *de novo* folate pathway (Auerbach et al., 2000). It has been reported that *GCH1* and *TH* (a rate-limiting enzyme for DA synthesis) can rescue feeding in dopamine-deficient mice (Sotak et al., 2005). *TPH2* is an appetite-related gene in the hypothalamus that exhibits the potential to regulate intestinal motility through serotonergic synapse and oxytocin signaling pathways (Miao et al., 2021). The mRNA expression of *TPH2* declined after the administration of sweeteners (an induced appetite) in broilers (Jiang et al., 2021). Therefore, modification of *GCH1, TPH2*, and *TH* expression in the hypothalamus may affect feeding mechanisms by increasing the metabolic flux and energy through the *de novo* folate biosynthesis pathway, thereby controlling appetite. This regulation may contribute to the differences in FE observed between low RFI and high RFI groups.

Further, we found that DEGs involved in tyrosine metabolism (*DDC, TH*, and *FAH*) were upregulated. *DDC* and *TH* are involved in DA synthesis via tyrosine metabolism (Wang et al., 2023). TH is the rate-limiting step for catecholamines (dopamine, norepinephrine, epinephrine) from tyrosine. Tryptophan hydroxylase (**TPH**), specifically TPH1 (in the periphery) and TPH2 (in the brain), are the rate-limiting enzymes for serotonin (an indoleamine) from tryptophan. Thus, TH dictates catecholamine levels, and TPH dictates serotonin levels, both being key neurotransmitters (Wickramasuriya and Lyte, 2026). DA is the most abundant catecholamine neurotransmitter in the brain, and it controls a wide range of cognitive functions, motor skills, food intake, endocrine regulation, and reward-based learning in mammals (Baik, 2021). In chickens, DA acts by connecting neuronal gene expression in the hippocampus and behavior in response to feeding conditions (Chen et al., 2021b). *FAH* is a catalyzing enzyme at the last step of the tyrosine metabolism pathway by converting fumarylacetoacetate into acetoacetate and fumarate. Therefore, the upregulation of *DDC, TH*, and *FAH* in the tyrosine metabolism pathway may lead to neurological or biochemical changes in the brain that alter feeding behavior (Moore et al., 2017), which may explain the differences in FE observed between the low-RFI and high-RFI groups.

The hypothalamic-pituitary-adrenal (**HPA**) axis is a crucial neuroendocrine system controlling feeding behavior in humans and animals (Cascino et al., 2023). Animals respond to stressors by increasing their metabolic rate, which enhances energy consumption and utilization through the regulation of glucocorticoid secretion via the HPA axis. In this study, *DDC, TPH2,* and tryptophan 2,3-dioxygenase (***TDO2***) were enriched in the tryptophan metabolism pathway, a pathway depending on glucocorticoid levels. *TDO2* plays a critical role in catalyzing the first and rate-limiting step of the kynurenine pathway (a major tryptophan metabolism pathway), while *DDC* and *TPH* influence the rate of serotonin synthesis (a major tryptophan metabolism pathway) (Neavin et al., 2018). In chickens, serotoninergic systems are involved in various physiological functional regulations, such as an inhibitory effect on feeding behavior, resulting in increased energy expenditure (Saadoun and Cabrera, 2002). Interestingly, *DDC TPH2* and *TDO2* were upregulated in the low-RFI chickens. Studies in rats have shown that thyrotropin-releasing hormone receptors (**TRHR**) are crucial for mediating the activating and antidepressant-like effects of TRH, potentially by stimulating serotonergic activity either indirectly or directly through complex intracellular signaling pathways (Chávez et al., 2022). Our previous study demonstrated that *TRHR3*, a pituitary gene involved in metabolic regulation, was significantly downregulated in the low-RFI KRC group (Sinpru et al., 2025). TRHR3 is activated by hypothalamic TRH and subsequently initiates intracellular signaling cascades mediated by RGS proteins, which play essential roles in modulating downstream metabolic processes (Cheng et al., 2023). Collectively, these findings suggest that low-RFI chickens may utilize energy more efficiently through enhanced regulation of serotonergic pathways, potentially involving TRHR-mediated signaling. Given the central role of the HPA axis in regulating feeding behavior and energy metabolism via stress-induced glucocorticoid secretion, future studies should incorporate a broader panel of stress-related hormones (e.g., corticosterone and adrenocorticotropic hormone) alongside neurotransmitters, and integrate multi-tissue analyses (e.g., hypothalamus and gut) within the same individuals. Additionally, experimental validation approaches, such as targeted gene expression assays or functional studies, are needed to confirm the causal roles of these pathways in FE.

It is important to recognize that FE is a multifaceted trait regulated by multiple physiological systems. In addition to central appetite regulation, intestinal function, nutrient digestibility, and peripheral nutrient sensing play key roles. The gastrointestinal tract is central to nutrient digestion, absorption, and sensing, and is a major determinant of FE. Recent studies in broiler chickens have demonstrated that intestinal morphology—including villus height, crypt depth, and absorptive surface area—as well as tight junction integrity, nutrient transporter expression, are strongly associated with FE variation (Beauclercq et al., 2018; Xiao et al., 2021). Birds with superior FE often exhibit enhanced intestinal barrier function, more efficient nutrient absorption, and optimized gut microbiota composition, which collectively contribute to improved nutrient utilization and reduced energy expenditure for maintenance (Beauclercq et al., 2018). Our research group has previously investigated the role of intestinal tissues in regulating FE in the KRC. Sinpru et al. (2021) conducted RNA–seq analysis of jejunal tissue in chickens with divergent RFI and identified DEG associated with nutrient transport, immune function, and metabolic processes. Similarly, Kaewsatuan et al. (2022) reported that duodenal transcriptomes differed between high- and low-FE birds, particularly in pathways related to tight junction integrity, glycolysis, and nutrient absorption. Kongthungmon et al. (2025) demonstrated that low-RFI chickens exhibited improved FE, accompanied by upregulation of tight junction-related genes (*ACTN1* and *TUBA3E*), increased ACTN1 protein expression, and more compact and structurally intact tight junctions. Collectively, these studies highlight the importance of intestinal function in determining FE in KRC, whereas the present study focuses on the hypothalamus to elucidate the integration of peripheral metabolic signals in FE regulation in slow-growing KRC.

It is well established that the gut–brain axis represents a bidirectional communication network, wherein peripheral signals influence hypothalamic gene expression and neurotransmitter synthesis, while central nervous system outputs—via autonomic innervation and neuroendocrine pathways—modulate gut motility, secretion, barrier function, and microbiota composition (Jiang et al., 2021; Bai et al., 2024). As noted previously, our research group has conducted transcriptomic analyses of jejunum (Sinpru et al., 2021) and duodenum (Kaewsatuan et al., 2022) tissues in the KRC. Although these studies did not specifically report enrichment of the three KEGG pathways identified in the present hypothalamic analysis (folate biosynthesis, tyrosine metabolism, and tryptophan metabolism), the metabolic genes identified in intestinal tissues are associated with nutrient metabolism processes. This suggests that related or tissue-specific pathways may also be active in the gut. Accordingly, future integrative multi-tissue transcriptomic analyses of the hypothalamus and gastrointestinal tract within the same individuals would be essential for elucidating the coordinated mechanisms underlying FE in KRC.

### Protein-protein interaction network of DEGs

**Fig. 3:**
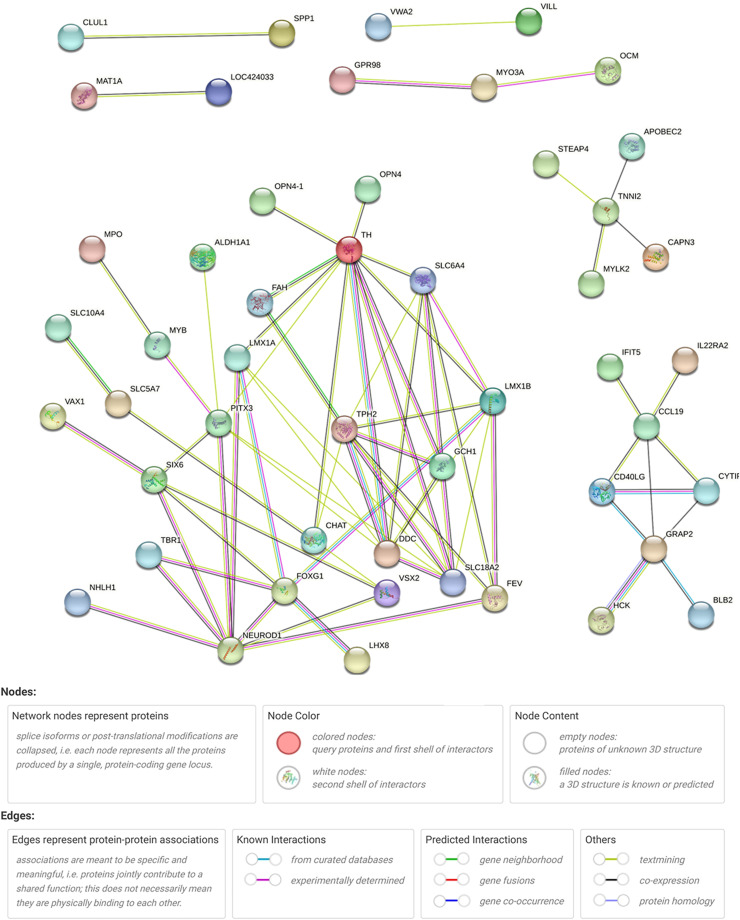
Protein-protein interaction network of differentially expressed genes (DEGs) in hypothalamic tissue between high-RFI and low-RFI groups. Nodes represent the DEGs identified with the coding gene symbol, colored nodes indicate the query proteins, and lines represent the connections between the proteins.

To gain insight into DEGs associated with FE, we conducted a protein-protein interaction (**PPI**) network analysis based on the STRING database to compare between groups (Fig. 3). The results revealed that five DEGs (*GCH1, TPH2, DDC, TH*, and *FAH*) were highly connected and potentially involved in modulating feeding behavior that affects FE. This finding is supported by a previous study suggesting that *GCH1* plays a critical role in regulating tetrahydrobiopterin synthesis through feedback inhibition, which influences metabolic pathways related to FE in the hypothalamus (Harada et al., 1993). *TPH2*, the rate-limiting enzyme in brain serotonin synthesis, is essential for serotonin production (Ottenhof et al., 2018). Although the regulatory mechanism of *TPH2* is not well understood in chickens, serotonin is known to influence homeostatic systems related to sleep, mood, and FI (Pereira et al., 2020). *TH* is the rate-limiting enzyme in DA synthesis, and DA levels in the hypothalamus are crucial for neuroendocrine functions that regulate appetite and FE (Lechan and Fekete, 2005). Moreover, interactions between DA and the HPA axis also regulate feeding responses, as the neural circuits controlling FI converge on the paraventricular nucleus, which contains neurons expressing corticotropin-releasing hormone and urocortin, both of which play roles in feeding behavior (Belda and Armario, 2009; Maniam and Morris, 2012). Our results indicate that variations in RFI between groups may be linked to the five genes identified through the PPI network, which were closely connected to other genes and highly associated with RFI.

### Gene validation using RT-qPCR

**Fig. 4:**
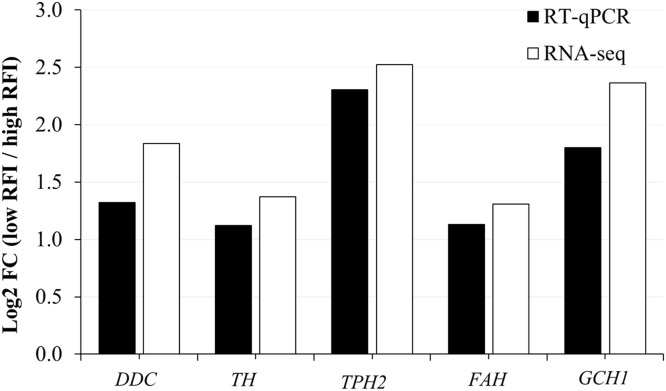
Relative expression trends between RNA-seq and RT-qPCR of five DEGs involved in modulating feed efficiency. *DDC*: l-DOPA decarboxylase; *TH*: tyrosine hydroxylase; *TPH2*: tryptophan hydroxylase 2; *FAH*: fumarylacetoacetate hydrolase; *GCH1*: GTP cyclohydrolase 1.

To confirm the differential expression values obtained from the statistical comparison of RNA-seq data, RT-qPCR was performed to validate the DEGs involved in the regulation of FE. Five genes (*GCH1, TPH2, DDC, TH*, and *FAH*) were selected based on their high connectivity and involvement in the network modulating feeding behavior. Fig. 4 demonstrates the expression patterns obtained from RT-qPCR were consistent with those observed in the RNA-seq for these five genes comparison between low-RFI and high-RFI groups. These findings confirmed that all five genes were differentially expressed between the two groups, highlighting the high reproducibility and reliability of the RNA-seq result in this study.

### Enriched KEGG pathway validation

**Fig. 5:**
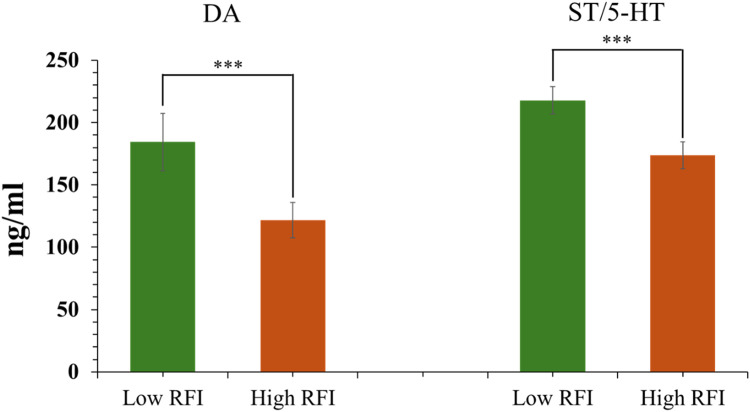
Comparison of plasma dopamine (DA) and serotonin/5-hydroxytryptamine (ST/5-HT) levels between low-RFI and high-RFI groups. Data were obtained from individual chickens (*n* = 10 per group) and presented as mean ± SEM. Statistical analysis was performed using Student’s t-test. *P < 0.05, ^⁎⁎^*P < 0.01*, ^⁎⁎⁎^*P < 0.001*).

*TPH2, DDC*, and *TH* genes are related to the three significantly enriched KEGG pathways of DEGs (folate biosynthesis, tyrosine metabolism, and tryptophan metabolism), and their involvement in these pathways was consistent with the concentrations of DA and ST/5-HT in plasma. The low-RFI group exhibited significantly higher plasma concentrations of DA and ST/5-HT than the high-RFI group (*P < 0.01*; Fig. 5). This finding is consistent with the trends observed in KEGG pathway enrichment.

Plasma concentrations of DA and ST/5-HT are key biological markers of neurotransmitter activity (Kim et al., 2023). In this study, the concordance between plasma DA and ST/5-HT levels and the involvement of key genes in enriched KEGG pathways suggests that monoamine (DA and ST/5-HT) synthesis and metabolism in the hypothalamus may contribute to differences in RFI in KRC. Notably, the upregulated genes *TPH2, DDC*, and *TH* in the low-RFI group were enriched in pathways related to folate biosynthesis, tyrosine metabolism, and tryptophan metabolism, consistent with the higher plasma concentrations of DA and ST/5-HT observed in this group. However, plasma neurotransmitter levels represent indirect measures and may not reflect hypothalamic synaptic activity due to peripheral production and the constraints imposed by the blood–brain barrier. Therefore, these findings provide supportive but not causal evidence for the involvement of neurotransmitter-related metabolic pathways in FE. Nevertheless, they offer a useful framework for future investigations into the molecular mechanisms underlying FE in slow-growing chickens.

## Conclusion

To our knowledge, this study is the first to report global gene expression in hypothalamic tissue underlying different FE in slow-growing chickens. In this study, the comprehensive analysis revealed that *GCH1, TPH2, DDC, TH,* and *FAH* are potential core genes that regulate differences in feeding behavior. Functional enrichment analysis of DEGs demonstrated that the FE-affected molecular mechanisms are mainly related to metabolic processes and transporter activity. KEGG pathway analysis identified folate biosynthesis, tyrosine metabolism, and tryptophan metabolism as significant pathways which related to FE of KRC. It is important to note that the upregulated genes (*TPH2, DDC,* and *TH*) in the low- RFI group were enriched in the KEGG pathway, which was consistent with the concentrations of DA and ST/5-HT in plasma. Although plasma levels are indirect and may not reflect hypothalamic synaptic neurotransmission, they provide supporting evidence for the metabolic pathways identified in this study and lay a theoretical foundation for future investigations into FE mechanisms in slow-growing chickens. The bulk hypothalamic tissue samples analyzed may dilute nucleus-specific signals. Future studies in nucleus-specific sampling or single-cell approaches would be beneficial to specific neuronal populations.

## CRediT authorship contribution statement

**Panpradub Sinpru:** Writing – review & editing, Writing – original draft, Visualization, Methodology, Conceptualization, Data curation. **Orapin Jantasaeng:** Writing – review & editing, Writing – original draft, Methodology, Conceptualization, Data curation, Visualization. **Phocharapon Pasri:** Writing – review & editing, Methodology. **Boonyarit Kamkrathok:** Writing – review & editing, Methodology. **Saknarin Pengsanthia:** Writing – review & editing, Software. **Tom E. Porter:** Writing – review & editing, Supervision, Investigation. **Wittawat Molee:** Writing – review & editing, Investigation, Supervision. **Amonrat Molee:** Writing – review & editing, Supervision, Funding acquisition, Conceptualization, Project administration, Resources.

## Disclosures

The authors declare no conflicts of interest.

## Appendix Supplementary materials

Image, image 1 dummy alt text

Image, application 2

Image, application 3

Image, application 4

Image, application 5

Image, application 6

## Footnotes

Supplementary material associated with this article can be found, in the online version, at doi:10.1016/j.psj.2026.107098.

## References

[bib0001] Arora S., Anubhuti (2006). Role of neuropeptides in appetite regulation and obesity – A review. Neuropeptides.

[bib0002] Auerbach G., Herrmann A., Bracher A., Bader G., Gütlich M., Fischer M., Neukamm M., Garrido-Franco M., Richardson J., Nar H., Huber R., Bacher A. (2000). Zinc plays a key role in human and bacterial GTP cyclohydrolase I. Proc. Natl. Acad. Sci..

[bib0003] Baéza E., Le Bihan-Duval E. (2013). Chicken lines divergent for low or high abdominal fat deposition: a relevant model to study the regulation of energy metabolism. Animal.

[bib0004] Bai H., Geng D., Xue F., Li X., Wang C., Wang C., Guo Q., Jiang Y., Wang Z., Bi Y., Chen G., Chang G. (2024). Gut–brain bidirectional determination in regulating the residual feed intake of small-sized meat ducks. Poult. Sci..

[bib0005] Baik J.H. (2021). Dopaminergic control of the feeding circuit. Endocrinol. Metab..

[bib0006] Banerjee P., Carmelo V.A.O., Kadarmideen H.N. (2020). Genome-wide epistatic interaction networks affecting feed efficiency in Duroc and Landrace pigs. Front. Genet..

[bib0007] Beauclercq S., Nadal-Desbarats L., Hennequet-Antier C., Gabriel I., Tesseraud S., Calenge F., Le Bihan-Duval E., Mignon-Grasteau S. (2018). Relationships between digestive efficiency and metabolomic profiles of serum and intestinal contents in chickens. Sci. Rep..

[bib0008] Belda X., Armario A. (2009). Dopamine D1 and D2 dopamine receptors regulate immobilization stress-induced activation of the hypothalamus-pituitary-adrenal axis. Psychopharmacol. (Berl).

[bib0009] Blouet C., Schwartz G.J. (2010). Hypothalamic nutrient sensing in the control of energy homeostasis. Behav. Brain Res..

[bib0010] Boswell T., Li Q., Takeuchi S. (2002). Neurons expressing neuropeptide Y mRNA in the infundibular hypothalamus of Japanese quail are activated by fasting and co-express agouti-related protein mRNA. Mol. Brain Res..

[bib0011] Cascino G., Chami R., Monteleone P. (2023). Eating Disorders.

[bib0012] Chávez, J., V. Alcántara-alonso, C. García-luna, P. Soberanes-chávez, D. Grammatopoulos, and P. De Gortari. 2022. Hypothalamic TRH mediates anorectic effects of serotonin in rats. 11:9. 10.1523/ENEURO.0077-22.2022 PMC9159524 35545425

[bib0013] Chen C., Su Z., Li Y., Luan P., Wang S., Zhang H., Xiao F., Guo H., Cao Z., Li H., Leng L. (2021). Estimation of the genetic parameters of traits relevant to feed efficiency: result from broiler lines divergent for high or low abdominal fat content. Poult. Sci..

[bib0014] Chen G., Hu X., Sugahara K., Chen J., Song X., Zheng H., Jiang Y., Huang X., Jiang J., Zhou W. (2007). Type-dependent differential expression of neuropeptide Y in chicken hypothalamus (Gallus domesticus). J. Zhejiang Univ. Sci. B.

[bib0015] Chen S., Yan C., Xiao J., Liu W., Li Z., Liu H., Liu J., Zhang X., Ou M., Chen Z., Li W., Zhao X. (2021). Domestication and feed restriction programming organ index, dopamine, and hippocampal transcriptome profile in chickens. Front. Vet. Sci..

[bib0016] Cheng X., Zhang H., Guan S., Zhao Q., Shan Y. (2023). Receptor modulators associated with the hypothalamus -pituitary-thyroid axis. Front. Pharmacol..

[bib0017] Cho C.E., Sánchez-Hernández D., Reza-López S.A., Huot P.S.P., Kim Y.-I., Anderson G.H. (2013). High folate gestational and post-weaning diets alter hypothalamic feeding pathways by DNA methylation in Wistar rat offspring. Epigenetics.

[bib0018] Guo R., Zeng T., Wang D., Zhao A., Zhou S., Huang Z., Chang Y., Sun H., Gu T., Chen L., Tian Y., Xu W., Lu L. (2024). Comparative analysis of the hypothalamus transcriptome of laying ducks with different residual feeding intake. Poult. Sci..

[bib0019] Hang T.T.T., Molee W., Khempaka S., Paraksa N. (2018). Supplementation with curcuminoids and tuna oil influenced skin yellowness, carcass composition, oxidation status, and meat fatty acids of slow-growing chickens. Poult. Sci..

[bib0020] Harada T., Kagamiyama H., Hatakeyama K. (1993). Feedback regulation mechanisms for the control of GTP cyclohydrolase I activity. Science.

[bib0021] Hou Y., Hu M., Zhou H., Li C., Li X., Liu X., Zhao Y., Zhao S. (2018). Neuronal signal transduction-involved genes in pig hypothalamus affect feed efficiency as revealed by transcriptome analysis. Biomed Res. Int..

[bib0022] Itafa B.T., Mohamed A.S., Abate W.H., Derseh M.A., Woldegiorgiss W.E. (2021). Effect of reciprocal crossing Koekoek and Sasso chickens on growth performance, feed efficiency, carcass yield, mortality rate, and genetic components. J. Appl. Poult. Res..

[bib0023] Izadnia H.R., Tahmoorespur M., Bakhtiarizadeh M.R., Nassiri M., Esmaeilkhanien S. (2019). Gene expression profile analysis of residual feed intake for Isfahan native chickens using RNA-SEQ data. Ital. J. Anim. Sci..

[bib0024] Jiang J., Qi L., Lv Z., Wei Q., Shi F. (2021). Dietary stevioside supplementation increases feed intake by altering the hypothalamic transcriptome profile and gut microbiota in broiler chickens. J. Sci. Food Agric..

[bib0025] Kaewsatuan P., Poompramun C., Kubota S., Yongsawatdigul J., Molee W., Uimari P., Molee A. (2022). Comparative proteomics revealed duodenal metabolic function associated with feed efficiency in slow-growing chicken. Poult. Sci..

[bib0026] Kim D., Pertea G., Trapnell C., Pimentel H., Kelley R., Salzberg S.L. (2013). TopHat2: accurate alignment of transcriptomes in the presence of insertions, deletions and gene fusions. Genome. Biol..

[bib0027] Kim J., Jung H., Yoon M. (2023). Relationship between plasma dopamine concentration and temperament in horses. Domest. Anim. Endocrinol..

[bib0028] Kongthungmon S., Pengsanthia S., Kamkrathok B., Kaewsatuan P., Molee W., Molee A. (2025). Impact of residual feed intake on jejunal tight junction morphology and gene expression in slow-growing Korat chickens. Poult. Sci..

[bib0029] Kosonsiriluk S., Sartsoongnoen N., Chaiyachet O., Prakobsaeng N., Songserm T., Rozenboim I., El Halawani M., Chaiseha Y. (2008). Vasoactive intestinal peptide and its role in continuous and seasonal reproduction in birds. Gen. Comp. Endocrinol..

[bib0030] Kubota S., Pasri P., Okrathok S., Jantasaeng O., Rakngam S., Mermillod P., Khempaka S. (2023). Transcriptome analysis of the uterovaginal junction containing sperm storage tubules in heat-stressed breeder hens. Poult. Sci..

[bib0031] Kuenzel W., Masson M. (1988).

[bib0032] Lechan R.M., Fekete C. (2005). Role of thyroid hormone deiodination in the hypothalamus. Thyroid.

[bib0033] Li W., Liu R., Zheng M., Feng F., Liu D., Guo Y., Zhao G., Wen J. (2020). New insights into the associations among feed efficiency, metabolizable efficiency traits and related QTL regions in broiler chickens. J. Anim. Sci. Biotechnol..

[bib0034] Li Z., Liu X., Zhang P., Han R., Sun G., Jiang R., Wang Y., Liu X., Li W., Kang X., Tian Y. (2018). Comparative transcriptome analysis of hypothalamus-regulated feed intake induced by exogenous visfatin in chicks. BMC Genomics.

[bib0035] Liu L., Yi J., Ray W.K., Vu L.T., Helm R.F., Siegel P.B., Cline M.A., Gilbert E.R. (2019). Fasting differentially alters the hypothalamic proteome of chickens from lines with the propensity to be anorexic or obese. Nutr. Diabetes.

[bib0036] Liu Y., Shen J., Yang X., Sun Q., Yang X. (2018). Folic acid reduced triglycerides deposition in primary chicken hepatocytes. J. Agric. Food Chem..

[bib0037] Livak K.J., Schmittgen T.D. (2001). Analysis of relative gene expression data using real-time quantitative PCR and the 2−ΔΔCT method. Methods.

[bib0038] Love M.I., Huber W., Anders S. (2014). Moderated estimation of fold change and dispersion for RNA-seq data with DESeq2. Genome Biol..

[bib0039] Maniam J., Morris M.J. (2012). The link between stress and feeding behaviour. Neuropharmacology.

[bib0040] Mao X., Cai T., Olyarchuk J.G., Wei L. (2005). Automated genome annotation and pathway identification using the KEGG Orthology (KO) as a controlled vocabulary. Bioinformatics.

[bib0041] Martin M. (2011). Cutadapt removes adapter sequences from high-throughput sequencing reads. EMBnet. J..

[bib0042] Miao Y., Mei Q., Fu C., Liao M., Liu Y., Xu X., Li X., Zhao S., Xiang T. (2021). Genome-wide association and transcriptome studies identify candidate genes and pathways for feed conversion ratio in pigs. BMC Genomics.

[bib0043] Moore M.E., Koenig A.E., Hillgartner M.A., Otap C.C., Barnby E., MacGregor G.G. (2017). Abnormal social behavior in mice with tyrosinemia type I is associated with an increase of myelin in the cerebral cortex. Metab. Brain Dis..

[bib0044] Neavin D.R., Liu D., Ray B., Weinshilboum R.M. (2018). The role of the aryl hydrocarbon receptor (AHR) in immune and inflammatory diseases. Int. J. Mol. Sci..

[bib0045] Neill J.D., Plotsky P.M., de Greef W.J. (1979). Catecholamines, the hypothalamus and neuroendocrinology — applications of electrochemical methods. Trends Neurosci..

[bib0046] Ottenhof K.W., Sild M., Lévesque M.L., Ruhé H.G., Booij L. (2018). TPH2 polymorphisms across the spectrum of psychiatric morbidity: a systematic review and meta-analysis. Neurosci. Biobehav. Rev..

[bib0047] Pereira G.R.C., Tavares G.D.B., de Freitas M.C., De Mesquita J.F. (2020). In silico analysis of the tryptophan hydroxylase 2 (TPH2) protein variants related to psychiatric disorders. PLoS One.

[bib0048] Poompramun C., Molee W., Thumanu K., Molee A. (2021). The significant influence of residual feed intake on flavor precursors and biomolecules in slow-growing Korat chicken meat. Anim. Biosci..

[bib0049] Saadoun A., Cabrera M. (2002). Effect of the 5-HT1A receptor agonist 8-OH-DPAT on food and water intake in chickens. Physiol. Behav..

[bib0050] Seong J., Kang J.Y., Sun J.S., Kim K.W. (2019). Hypothalamic inflammation and obesity: a mechanistic review. Arch. Pharm. Res..

[bib0051] Sinpru P., Riou C., Kubota S., Poompramun C., Molee W., Molee A. (2021). Jejunal transcriptomic profiling for differences in feed conversion ratio in slow-growing chickens. Animals.

[bib0052] Sinpru P., Jantasaeng O., Pasri P., Kamkrathok B., Kaewsatuan P., Pengsanthia S., Molee W., Rau A., Porter T.E., Tixier-Boichard M., Molee A. (2025). Transcriptome profiling of the pituitary gland reveals candidate genes for divergent feed efficiency in slow-growing chickens. Ital. J. Anim. Sci..

[bib0053] Sintubin P., Greene E., Collin A., Bordas A., Zerjal T., Tesseraud S., Buyse J., Dridi S. (2014). Expression profile of hypothalamic neuropeptides in chicken lines selected for high or low residual feed intake. Neuropeptides.

[bib0054] Sohn J.W. (2015). Network of hypothalamic neurons that control appetite. BMB Rep..

[bib0055] Sotak B.N., Hnasko T.S., Robinson S., Kremer E.J., Palmiter R.D. (2005). Dysregulation of dopamine signaling in the dorsal striatum inhibits feeding. Brain Res..

[bib0056] Takele Assefa A., Vandesompele J., Thas O. (2020). On the utility of RNA sample pooling to optimize cost and statistical power in RNA sequencing experiments. BMC Genomics.

[bib0057] Teufel M., Sobetzko P. (2022). Reducing costs for DNA and RNA sequencing by sample pooling using a metagenomic approach. BMC Genomics.

[bib0058] Tola F.S. (2024). The concept of folic acid supplementation and its role in prevention of neural tube defect among pregnant women: PRISMA. Medicine.

[bib0059] Wang H., Xia P., Lu Z., Su Y., Zhu W. (2023). Time-restricted feeding affects transcriptomic profiling of hypothalamus in pigs through regulating aromatic amino acids metabolism. J. Sci. Food Agric..

[bib0060] Wickramasuriya S.S., Lyte J.M. (2026). Neurochemical metabolic pathways display regional and age-specific expression patterns in the chicken intestinal tract. Poult. Sci..

[bib0061] Xiao C., Deng J., Zeng L., Sun T., Yang Z., Yang X. (2021). Transcriptome analysis identifies candidate genes and signaling pathways associated with feed efficiency in Xiayan chicken. Front. Genet..

[bib0062] Yu X., Liu R., Zhao G., Zheng M., Chen J., Wen J. (2014). Folate supplementation modifies CCAAT/enhancer-binding protein α methylation to mediate differentiation of preadipocytes in chickens. Poult. Sci..

[bib0063] Zeng T., Chen L., Du X., Lai S.J., Huang S.P., Liu Y.L., Lu L.Z. (2016). Association analysis between feed efficiency studies and expression of hypothalamic neuropeptide genes in laying ducks. Anim. Genet..

[bib0064] Zhuo Z., Lamont S.J., Lee W.R., Abasht B. (2015). RNA-seq analysis of abdominal fat reveals differences between modern commercial broiler chickens with high and low feed efficiencies. PLoS One.

