# Supplementary figures and images for: RNA-seq reveals differentially expressed genes involved in catecholamine synthesis and metabolism in the hypothalamus affecting divergent residual feed intake in slow-growing Korat chickens
Source: Poult Sci. 2026 May 8;105(8):107098. doi: 10.1016/j.psj.2026.107098 (PMC13223840; doi:10.1016/j.psj.2026.107098)

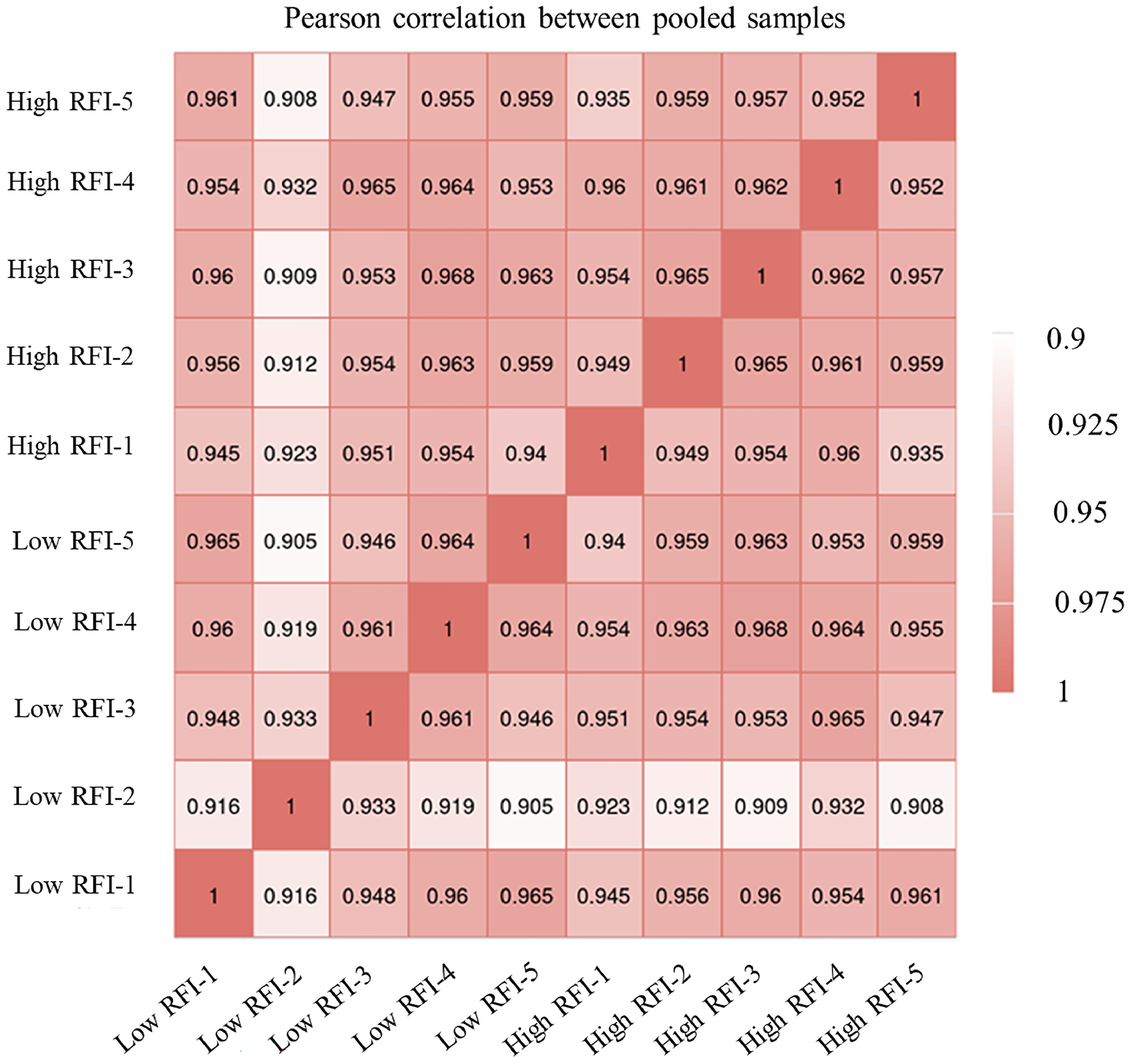

Supplement: Supplementary file 1 [file mmc1.jpg]
